# Supplementary material for: Iterative improvement in the automatic modular design of robot swarms
Source: PeerJ Comput Sci. 2020 Dec 7;6:e322. doi: 10.7717/peerj-cs.322 (PMC7924708; doi:10.7717/peerj-cs.322)
Supplement: Supplemental Information 3 [file peerj-cs-06-322-s003.zip › argos3/doc/api/standalone/a00466.html]

ARGoS: plugins/robots/foot-bot/simulator/footbot\_gripper\_default\_actuator.cpp File Reference


- Main Page
- Related Pages
- Namespaces
- Classes
- Files

- File List
- File Members

# plugins/robots/foot-bot/simulator/footbot\_gripper\_default\_actuator.cpp File Reference

`#include "footbot_gripper_default_actuator.h"`  
`#include <argos3/core/utility/logging/argos_log.h>`  
`#include <argos3/core/simulator/entity/composable_entity.h>`  

Include dependency graph for footbot\_gripper\_default\_actuator.cpp:

Go to the source code of this file.

|  |  |
| --- | --- |
| Namespaces | |
| namespace | argos |

|  |  |
| --- | --- |
|  | The namespace containing all the ARGoS related code. |

| Functions | |
|  | REGISTER\_ACTUATOR (CFootBotGripperDefaultActuator,"footbot\_gripper","default","Carlo Pinciroli [ilpincy@gmail.com]","1.0","The footbot gripper actuator.","This actuator controls the foot-bot gripper. For a complete description of its\n""usage, refer to the ci\_footbot\_gripper\_actuator.h file.\n\n""REQUIRED XML CONFIGURATION\n\n"" <controllers>\n"" ...\n"" <my\_controller ...>\n"" ...\n"" <actuators>\n"" ...\n"" <footbot\_gripper implementation=\"default\" />\n"" ...\n"" </actuators>\n"" ...\n"" </my\_controller>\n"" ...\n"" </controllers>\n\n""OPTIONAL XML CONFIGURATION\n\n""None.\n","Usable") |

---

## Function Documentation

|  |  |  |  |  |
| --- | --- | --- | --- | --- |
| REGISTER\_ACTUATOR | ( | CFootBotGripperDefaultActuator | , |  |
|  |  | "footbot\_gripper" | , |  |
|  |  | "default" | , |  |
|  |  | "Carlo Pinciroli " | [ilpincy @gmail.com], |  |
|  |  | "1.0" | , |  |
|  |  | "The footbot gripper actuator." | , |  |
|  |  | "This actuator controls the foot-bot gripper. For a complete description of its\n"" | *usage*, |  |
|  |  | refer to the ci\_footbot\_gripper\_actuator.h file.\n\n""REQUIRED XML CONFIGURATION\n\n""< controllers >\n""...\n""< my\_controller...>\n""...\n""< actuators >\n""...\n""< footbot\_gripper implementation=\"default\" />\n"" ...\n"" </actuators>\n"" ...\n"" </my\_controller>\n"" ...\n"" </controllers>\n\n""OPTIONAL XML CONFIGURATION\n\n""None.\n" | , |  |
|  |  | "Usable" |  |  |
|  | ) |  |  |  |

---

Generated on 10 Jul 2018 for ARGoS by 
 1.6.1
